# The Effects of UPcomplish on Office Workers’ Sedentary Behaviour, Quality of Life and Psychosocial Determinants: A Stepped-Wedge Design

**DOI:** 10.1007/s12529-022-10054-0

**Published:** 2022-01-31

**Authors:** Nathalie M. Berninger, Guy Plasqui, Rik Crutzen, Robert A. C. Ruiter, Gerjo Kok, Gill A. Ten Hoor

**Affiliations:** 1 Department of Work and Social Psychology, Maastricht University, P.O. Box 616, 6200 MD Maastricht, The Netherlands; 2 Department of Human Biology and Movement Sciences, Nutrition and Translational Research in Metabolism, Maastricht University, P.O. Box 616, 6200 MD Maastricht, The Netherlands; 3 Department of Health Promotion, CAPHRI, Maastricht University, P.O. Box 616, 6200 MD Maastricht, The Netherlands

**Keywords:** Sedentary behaviours, Intervention mapping, Quality of life, Vitality, Office workers, Compositional data approach

## Abstract

**Background:**

Sedentary behaviour (SB) affects cardiometabolic health and quality of life (QoL). We examine the effects of UPcomplish, a 12-week data-driven intervention, on SB, QoL and psychosocial determinants among office workers.

**Methods:**

Participants were recruited via judgement sampling. Five groups starting with time-lags of 7 weeks (*n* = 142, 96 females) received 14 feedback messages (FBMs) which were tailored to SB patterns, goals and hurdles. Participants received questionnaires at the beginning, middle and end of the intervention and wore an accelerometer measuring SB, operationalized as proportions (compositional data approach, CoDA) and summed squared sitting bouts (SSSB). We used linear mixed-effects models with random intercepts for weeks (between-subjects) and individuals (within-subjects).

**Results:**

UPcomplish did not reduce SB. Within-subjects compared to baseline, FBM #3 (βCoDA = 0.24, *p* < .001, 95% CI [0.15, 0.33]; βSSSB = 20.83, *p* < .001, 95% CI [13.90, 27.28]) and #4 (βCoDA = 0.20, *p* < .001, 95% CI [0.11, 0.29]; βSSSB = 24.80, *p* < .001, 95% CI [15.84, 33.76]) increased SB. QoL was unaffected. Perceived susceptibility was lower after FBMs #6 to #8 (βbetween = − 0.66, *p* = .04, 95% CI [− 1.03, − 0.30]; βwithin = − 0.75, *p* = .02, 95% CI [− 1.18, − 0.32]). Within-subjects, intentions to sit less were higher after FBMs #1 to #5 (1.14, *p* = .02, 95% CI [0.61, 1.66]). Improvements in determinants and in SB were not associated, nor were improvements in SB and in QoL.

**Conclusions:**

Compared to VitaBit only, UPcomplish was not beneficial. Environmental restructuring might be superior, but detailed analyses of moderators of effectiveness are needed.

**Supplementary Information:**

The online version contains supplementary material available at 10.1007/s12529-022-10054-0.

## Introduction

Type 2 diabetes, cardiovascular disease [1, 2] and mental health problems [3] are potential consequences of sedentary behaviours (SB), which include sitting, lying or reclining behaviours (excl. sleeping) that exhibit low energy expenditures [4]. Except for amounts of more than 10 h, not the sitting time per se seems to be detrimental, but a pattern with bouts of long, uninterrupted SB [5, 6]. Indeed, regular SB interruptions of standing and light activity with the same energy expenditure as single bouts of MVPA seem to be at least equally effective in reducing cardiometabolic risk [7].

The mechanisms of how SB affects physical and mental health are complex. During SB, the muscles of the lower limbs are static, which reduces blood flow, downregulates endothelial functions, and increases inflammation [1]. These aspects yield physical problems but also impact brain health and quality of life (QoL) [8–10]. For example, SB involves low muscle contractions suppressing the lipoprotein lipase in red muscle fibres [11]. Ineffective triglyceride metabolism and visceral fat increase insulin resistance and reduce binding of leptin in the hypothalamus and hippocampus, which is responsible for synaptic plasticity [8]. Moreover, cerebral blood flow and the release of neurotrophines are reduced during SB [9]. These mechanisms might impair cognitive functioning, vitality and thus performance [10]. Furthermore, prolonged SB increases the pressure on the intervertebral disks and weakens posterior lumbar structures, explaining its link to increased intensities of lower back pain [12, 13], and to neck and upper extremity musculoskeletal symptoms [14]. Lastly, despite a lack of clarity about the mechanisms, SB has been linked to stress and mental health problems [15, 16].

Since modernization yielded a higher prevalence of office work including about 60% of SB, interventions effective in reducing SB among office workers are needed [17]. Interventions that have been effective seem to include either environmental changes such as standing desks or personal coaches in addition to persuasion techniques [18–20]. Using technology to communicate tailored feedback and advice might be a more cost-efficient way than regular personal coaching [21–23]. Yet, purely computer-tailored SB interventions having shown reductions in workplace SB did not find these effects when combining working and leisure time SB [24–26]. Therefore, a personal coach providing tailored but automated feedback might be the optimal mixture of a low-cost yet personal intervention.

We applied the intervention mapping (IM) protocol to systematically develop a data-driven SB intervention aimed at a reduction of SB among office workers: UPcomplish [28, 29]. Workplace physical activity interventions that have been developed with IM have yielded promising effects [30–32]. The development of UPcomplish using evidence from the literature and from theories (e.g. reasoned action approach [33]) is described elsewhere [27]. Shortly, the problem of SB was refined, and behavioural outcomes, and performance objectives (i.e. sub-behaviours), were formulated. Important and changeable psychosocial determinants (e.g. attitude, perceived behavioural control, PBC) were linked to the performance objectives. We selected evidence-based behaviour change methods and translated them into practical applications by the help of parameters for use and by considering the change objectives (i.e. change needed in the determinants to realize the performance objectives) [34, 35]. For example, the method “consciousness raising” can help to change “attitude”, which will likelier yield a decision to reduce SB if the rise of awareness (i.e. of negative consequences of SB), is rapidly followed by an increase in self-efficacy [29, 35]. Pre-tests and a pilot test facilitated further refinement and the automation of the 14 feedback messages (FBMs) [27]. The main component was “UPcomplish” consisting of 14 FBMs, which automatically tailored to participants’ SB patterns, and were send by a coach. The FBMs tackled self-efficacy, attitude, perceived social support (PSS), perceived susceptibility (PS), and normative beliefs through implementation intentions, monitoring, tailored feedback, and motivational support [36]. The second component, VitaBit, served as monitoring toolkit providing information, at which point in time participants were sitting, standing, or moving [37].

**Fig. 1:**
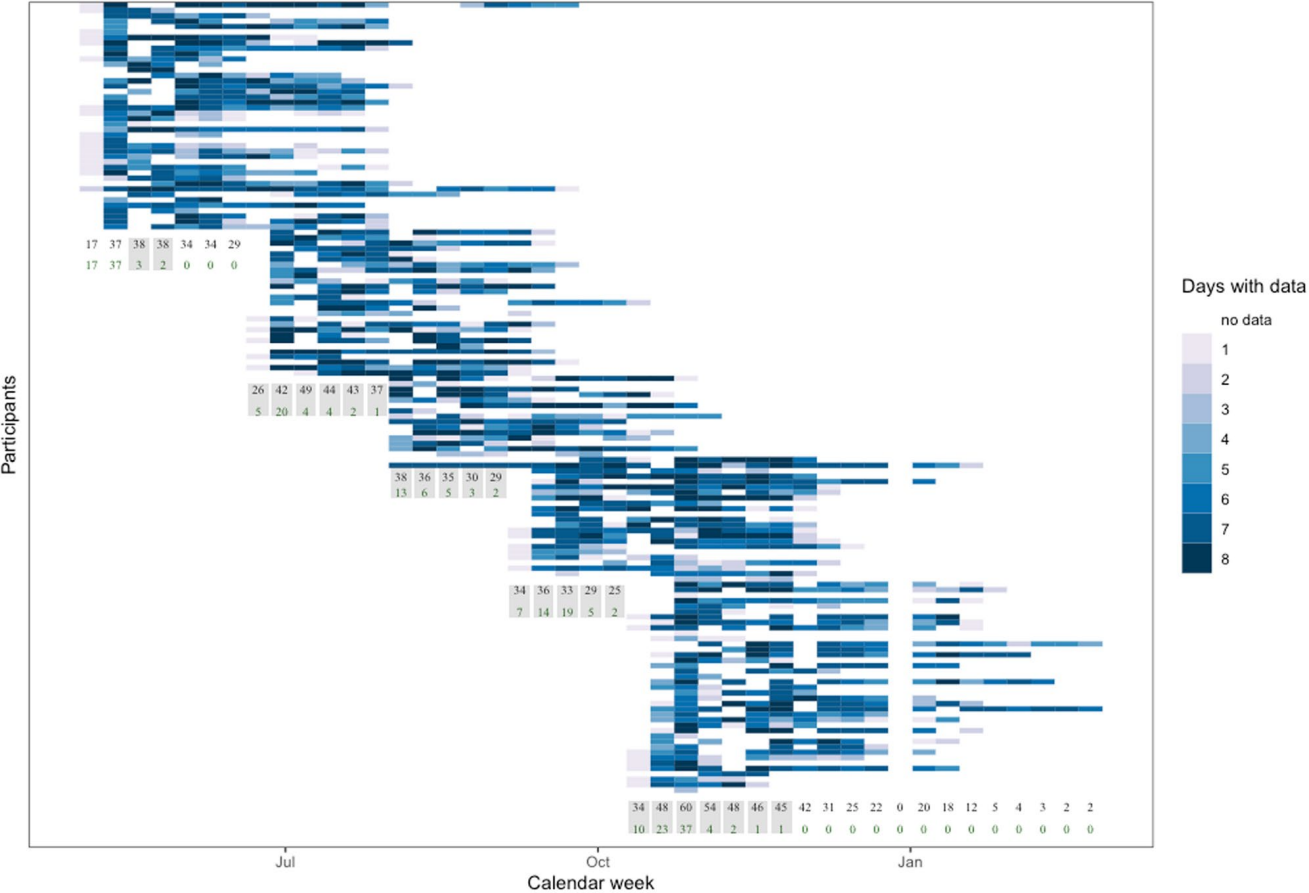
Flowchart of the stepped-wedge trial of the UPcomplish intervention. The black numbers indicate per week, how many participants provided data; the green numbers how many participants were in their baseline phase during the concerning week. Weeks with both baseline and intervention data, which are relevant for the between-subjects’ comparisons, are marked with grey cuboids

The primary objective of this study was to investigate the effects of UPcomplish on objectively measured SB, self-reported QoL (i.e. perceived performance, stress, pain, emotional well-being, EWB, vitality), and psychosocial determinants (i.e. attitude, PSS, PBC, PS, intention). Between and within subjects, we expected UPcomplish to reduce the daily proportion of SB and prolonged sitting when compared to monitoring only phases, i.e. baseline periods. Furthermore, we expected improvements in QoL and in the psychosocial determinants. The secondary objective was to explore correlates between the variables being assessed. We expected that the psychosocial determinants would be correlated with SB, and that SB would be correlated with QoL. We chose a stepped-wedge design (Fig. 1) above a parallel randomized control trial to reduce the burden for participants in a potential waiting control group (e.g. compliance), to increase statistical power (i.e. groups act as both control and intervention group; continuously measured SB) [38], and to gather seasonal spread data.

## Methods

This study was pre-registered: NL7503 (https://www.trialregister.nl/trial/7503). The intervention protocol can be found in [27]. The cleaned raw data and additional material are fully disclosed in the supplementary materials (https://osf.io/qzp9m/?view_only=30ada8d6fc0e4ac19a1610b8901f9f96). We adhere to the Consolidated Standards of Reporting Trials (CONSORT) checklist of information to include when reporting a stepped wedge cluster randomized trial [39].

### Study Design and Sample

Five intervention groups (Fig. 1) of maximum 40 participants started with time lags of about 7 weeks (exact duration depended on holidays and availabilities). Participants were eligible to take part if they were able to stand and walk, and willing to download the VitaBit app on their smartphones (at least Android 4.3 or iOS 8.1). Furthermore, only people who defined themselves as office workers and who understood the German language could participate. If any of the inclusion criteria were not met, participants were excluded.

All groups participated in a kick-off (incl. generic information on SB and the health consequences, which in itself has not been linked to changes in SB before [40]). To create a personal atmosphere and considering time constraints but still be efficient, the kick-offs were held for different sub-groups (i.e. companies) with a maximum of 15 and a minimum of 5 participants. The sub-groups were continuously recruited starting in one of the upcoming kick-offs that they would be available. If many participants per group dropped out, sub-groups were merged to still allow for group activities (e.g. challenges, group report). A baseline week after the kick-off session served as the control condition, where participants wore the VitaBit without receiving any tailored FBMs. After the baseline week, everyone received the intervention consisting of 14 FBMs. Each FBM targeted specific psychosocial determinants and change objectives, but were tailored to individual behaviours and goals (e.g. a suggestion of a goal targeted PBC but contained different goals per individual).

All individuals were compared to their baseline week (within-subjects’ comparisons). Some of the calendar weeks included participants being at their baseline and participants having already received the intervention (between-subjects’ comparisons). To test whether a certain dose of the intervention might be necessary to be effective, the reception of different amounts of FBMs being sent were analysed in separate regression models. For example, in calendar week 27, 42 participants had worn the VitaBit device. Among these, 20 participants had still been in their baseline week, and 12 had just received FBM #5. When analysing the between-subjects’ effect of FBM #5, these 12 participants were compared to the 20 participants being in their baseline week. To increase statistical power, the FBMs were aggregated when analysing the effects on psychosocial determinants and QoL, since these were assessed via surveys at three times: baseline (T0), week 6 (T1) and at the end of the intervention (T2).

For the time of the evaluation (May 2019–January 2020), we had 200 VitaBit sensors to our disposal. With an anticipated drop-out rate of 20% and five intervention groups (32 participants per group after drop-out and the middle group providing data for both baseline and intervention), we expected to end with a sample size of 192, which would reveal sufficient power according to our sample size planning [27].

Participants could refuse participation at all times, without giving a reason. Yet, most participants that dropped out gave a reason (e.g. technical problems, time constraints). This study and its consent procedure was approved by the Ethics Review Committee of the Faculty of Psychology and Neuroscience, Maastricht University, the Netherlands [ERCPN- 188_11_02_2018]. The trial was pre-registered under: NL7503.

### Procedure

#### Recruitment

The UPcomplish coach (psychologist employed by VitaBit software) was trained by the intervention developers. We contacted as many German companies as resources allowed (selected via judgement sampling; number not noted) via contact persons, personal conversations, emails, and phone calls and distributed the flyers. These included information on what would be expected from participants (e.g. downloading the app), and how much time participation would require (i.e. 1 h kick-off, 14 × 2 min feedback, and 3 × 20 min surveys). Additionally, it included information about inclusion criteria (e.g. being a desk worker, able to walk) and about the benefits one could expect from participation (e.g. vitality through a reduction of SB). As soon as the management of the companies agreed, a date for the kick-off was arranged. The participants were invited via email and received an instruction on the creation of a VitaBit account, the information sheet and the informed consent.

#### Kick-Off and Measurement Time-Points

For the kick-offs, the coach visited the participants in their companies. The duration depended on the size of the concerning sub-group and the number of questions (35 to 60 min). The introduction including an estimation of participants’ daily sitting times (on workdays and on days off) was followed by an explanation about the consequences of SB and by information about how UPcomplish could help them to reduce SB. Afterwards, participants were told to choose a realistic but challenging goal (e.g. sitting for a max. of 8 h per day), which would be adapted after the baseline week if necessary. These goals served as orientation for the participants and as basis to give first tailored advice.^1^ The coach explained the functionalities of the VitaBit toolkit and clarified questions. Participants were informed that they would receive an individual and a group report (i.e. at company level) and a 50€ VitaBit voucher as compensation for their participation. Furthermore, they received information on the purpose of study, detailed information on the participation procedure, data management, and potential benefits, before written informed consent was obtained. The VitaBit devices were distributed and connected via Bluetooth with the smartphone app. Participants who were not able to make it to the kick-off received an email with the information and hand-outs. At the end of the kick-off, the participants started wearing the VitaBit device. The week after the kick-off served as baseline and involved the first survey on QoL and on determinants. Afterwards, participants received the intervention including the second survey in week 6 and the last survey after the intervention. The 4 weeks after the intervention served as follow-up measurement and participants received their compensation, before the devices were collected.

### Intervention

The protocol of the intervention and the link between the FBMs and the psychosocial determinants are described elsewhere [27]. For each FBM, the authorized coach downloaded the raw data (pseudonymized IDs and SB) from the server. The data were imported into R statistical software where the code cleaned and transformed them in such a way, that it provided the coach with tailored FBMs for all participants (either with the next FBM or with a reminder). The coach delivered the FBMs through the participants’ preferred communication-channel (WhatsApp or email). Participants received two FBMs per week, which was reduced to one FBM per week as of week 6 (see Table 1 for an overview of the FBMs). The FBMs were not delivered, if a participant had dropped out, was on a holiday (if they indicated to pause for their holidays), or if not enough data were available (i.e. depending on the FBM less than 1 to 3 days à 6 h of data). If insufficient data were available on a feedback day, instead of receiving the next FBM, they received a reminder to synchronize their data or were asked if they still participated (maximum two reminders in a row). In case a participant received a reminder or did not receive any message, the concerning FBM was sent in the week after and the following FBMs were delayed also. FBMs #13 (*Competing colleagues*) and #14 (*Tips how to keep new habits in the future*) were not delayed and sent to all active participants in the last two weeks. Therefore, if participants missed two FBMs, they received FBMs #1 to #10, #13 and #14. Based on the baseline data and the goals from the kick-off meeting, the goals were adapted if necessary and broken down into graded sub-goals. In addition to feedback about goals, participants received tailored FBMs about their SB pattern (e.g. *On Tuesday noons between 11:00 and 14:00[…], your sitting periods seem to be specifically long. Here is a tip […]*), questions about individual hurdles (e.g. *What hinders you most when reducing your sitting behaviour? Is it habits, lack of time, […]?*), and tailored tips to overcome personal hurdles. The FBMs also included challenges in biweekly circles. The last two weeks focused on sustaining new behaviours by the help of if–then-plans and finding a buddy. The second intervention component was the VitaBit app including tools to monitor SB, such as a “Vitality score” (0 = unhealthy SB pattern, 100 = healthy SB pattern), the current amount of SB, and goal achievements.

**Table 1:** Overview of the FBMs of the UPcomplish intervention

		Delivery^a^
#1	*Goal adaption and sub-goals*	Monday, week 2
#2	*Feedback sitting pattern and first challenge*	Thursday, week 2
#3	*What are your hurdles to sit less?*	Monday, week 3
#4	*Tips how to overcome hurdles*	Thursday, week 3
#5	*Feedback on goal achievement*	Monday, week 4
#6	*Feedback sitting pattern and second challenge*	Thursday, week 4
#7	*Goal adaption and long-term goal*	Monday, week 5
#8	*Feedback on sitting pattern and on goal achievement*	Thursday, week 5
#9	*Feedback goal achievement and third challenge*	Thursday, week 6
#10	*Did your hurdles change?*	Thursday, week 7
#11	*Feedback goal achievement and last sub-goal*	Thursday, week 8
#12	*Feedback sitting pattern and fourth challenge*	Thursday, week 9
#13	*Competing colleagues*	Thursday, week 10
#14	*Tips how to keep new habits in the future*	Thursday, week 11

^a^Point in time if device is worn at all points in time

### Measures

#### Behavioural Measurements

Physical behaviour was continuously measured using accelerometery [41]. The VitaBit sensor (3.9 × 1.4 × 0.85 cm, 4.8 g) was worn in trouser pockets or at the thigh (i.e. attached with a magnet). The battery life of the device is at least 30 days, and it shows sensitivity and specificity values of 85.7% and 91.2%, respectively, for SB [37]. The device deploys a sampling rate of 33 Hz and an output data rate of 30 s. Data are stored on the device for at least 30 days and can be synchronized with the VitaBit app via Bluetooth. Via Internet, the data are sent to a back-end server, where they are processed and stored (pseudonymized) in a time series database. An authorized coach can download them from the portal. The data cleaning procedure to retrieve the SB variables is described under the “Data Preparation” section.

The performance objectives (e.g., participants create a VitaBit account) were retrieved from behavioural observations. These will be analysed as potential moderators of effectiveness in a future article and are described in more detail elsewhere [27].

#### Online Survey

The survey was distributed at baseline (T0), after 6 weeks (T1), and after the intervention (T2). Sociodemographic and job-related variables were measured at T0, intervention characteristics (e.g., acceptability, understandability) at T2. Psychosocial determinants and QoL were measured at all three time points. We translated the Individual Work Performance Questionnaire into German using back-translation [42]. As indicators for reliability, we present Omegas (ω) if more than 2 items were used for a construct, and Pearson correlations (*r*) if only two items were used [43, 44].

VitaBit obtained gender, age, education, height, weight, and job-related variables when participants created the account. They could choose between 8 educational degrees (e.g., Master’s degree), between 29 job titles (e.g., sales, administrative), between 17 company industries (e.g., service, finance), and between different team sizes. At T0, they were asked about the usual number of workdays per week (from 1 to 7; 1 item), employment status (full-time/part-time; 1 item) and job tasks (5 items). These included phone calls, computer work, desk work, having meetings, and travelling/visiting clients, e.g. “How much—on average per day (in %)—do you estimate that you spend on the following tasks? Phone calls?” [45].

Task and contextual performance were assessed by subscales of the Individual Work Performance Questionnaire (seldom = 0 to always = 5). Task performance (5 items; ω = 0.72) refers to the ability of performing the tasks being required for the job, operationalized as work quantity and quality or job skills, e.g. “During the last week, I was able to perform my work well with minimal time and effort”. Contextual performance (9 items; ω = 0.57) refers to the organizational, social, or psychological requirements facilitating functioning at work, such as investing effort or cooperating, e.g. “I took on extra responsibilities.” [46]. Stress perception was administered by the Perceived Stress Scale (10 items; e.g. “How often have you felt nervous and ‘stressed’?”; ω = 0.89) [47, 48]. Bodily pain (2 items; e.g. “How much bodily pain have you had?”; r = 0.85), EWB (5 items; e.g. “How much of the time have you been a happy person?”; ω = 0.83), and vitality (4 items; e.g. “How much of the time did you have a lot of energy?”; ω = 0.86) were assessed by subscales of the SF-36 [49].

We assessed the psychosocial determinants by questions about how much they agreed with certain statements. The items for attitudes (6 items; e.g. “[…] walking around at work is healthy”; ω = 0.62), PSS (2 items; e.g. “[…] walking around at work is encouraged by my colleagues”; *r* = 0.62), PBC (4 items; e.g. “I am sure that I can […] walk around at work, even though I feel bad, tired, tense or depressed”; ω = 0.70), and intention (2 items; e.g. “Are you planning to interrupt long sitting periods at work with […] walking breaks?”; *r* = 0.43) were based on former evaluation papers [45]. Additionally, we assessed PS, which refers to the belief to be at risk of getting a disease (2 items; e.g. “My daily sitting time is more compared to what is recommended.”; *r* = 0.72) [50, 51].

### Data Preparation

Activity, survey data, and dates of received FBMs were merged using pseudonymized user identifiers. Since the three physical behaviour levels are multicollinear (e.g. more sitting results in less standing and walking), we applied a compositional data analysis approach (CoDA) to transform them into non-interdependent variables [52]. We transformed the proportions of the three physical behaviours in relation to the entire waking day (i.e. when the device was worn) into isometric log-ratios by adjusting for the proportions spent in the other two behaviours (i.e. ${z1}_{sitting}=\sqrt{2/3 } \mathrm{ln}(Sitting\%/\sqrt{Standing\% x Activity\%} ))$ [53]. To analyse the effects on prolonged sitting, we used the sum of the squared sitting bouts (SSSB) [27]. To weigh longer sitting bouts more than shorter bouts, daily sitting bouts are squared before being summed up ($SSSB=\sum_{0}^{n}{SitBout}_{i}^{2}$). Afterwards, the data were cleaned to retain only those days that a participant collected enough data. Since there is always a trade-off between the retention of a high number of days and the retention of long days [54], we inspected the data by a plot: how many days would be retained for which daily wear time cut-off. Each stricter wear time cut-off resulted in fewer analysable days. The wear time cut-off of 8 h per day seemed to be a turning point (see Appendix A): each additional hour of required wearing time drastically reduced the number of available days. Therefore, only days with at least 8 h of VitaBit data were retained. Holidays were excluded from the analyses.

We created 14 variables with Boolean values representing whether the concerning FBM was already received at the concerning point in time, e.g. FBM_4_Received (TRUE/FALSE/NA). These variables were FALSE, if a participant had not received any FBM (i.e., baseline week), and TRUE, if a participant had just received the concerning FBM (e.g. #4). The variables were NA, if a participant had received more or less FBMs than the concerning FBM. The NAs were removed in the regression models to disentangle intervention effects from all other FBMs. Therefore, the reception of FBM #4 (i.e. FBM_4_Received = TRUE) was compared against baseline (i.e. FBM_4_Received = FALSE). For each individual, the days were averaged by FBM, for example, all days after FBM #4 but before #5 were averaged. Outliers were excluded using the Mahalanobis distance method (generalized squared distance), which is used for multidimensional data and is defined as the distance of each point (row in the matrix) from a distribution, normalized by the standard deviation, and adjusted by the covariances of the variables [55].

### Data Analyses

We used histograms and QQ plots to assess the distribution of the data. Non-normally distributed variables were reported as medians and interquartile ranges (IQRs), normally distributed variables as means and standard deviations (SDs), and categorical variables as absolute numbers and percentages.

To examine the between-subjects’ effects of UPcomplish on SB, QoL and psychosocial determinants, we used linear mixed-effects models with random intercepts for calendar week (which was dropped for QoL and the determinants, due to singularity). For comparability, all outcome variables were centred around the baseline sub-group means, and non-normally distributed variables (i.e. SSSB) were transformed to a normal distribution using square roots. For assessing within-subjects’ effects, the outcome variables (SB, QoL, and determinants) were centred around calendar week means (of baseline data), before deploying linear mixed-effects models with random intercepts for user identifier.

As a post hoc analysis, to analyse whether within-subjects’ improvements (centred around calendar week means) in determinants, in SB, and in QoL were associated, we conducted pairwise Pearson correlations. Changes were calculated by subtracting the values at T0 from the values at T2 (survey variables), and by calculating average improvements (SB). To calculate SB improvements, the SB data at one point in time (e.g. FBM #3) was subtracted by the SB data at the previous point in time (e.g. FBM #2), and divided by the previous point in time (e.g. FBM #2). All resulting differences were then averaged on the individual level.

Tests for statistical significance were two-sided with an alpha of 0.05, which was corrected using the Benjamini–Hochberg procedure [56, 57]. We used R version 3.4.1 to clean and analyse the data. We used backwards elimination to select the covariates (retention if *p* < 0.20). As potential covariates we included age, gender, body mass index (BMI), education, work tasks, employment status, and weekly working days. As a result, we controlled for gender (locked in the model) when analysing the intervention effects on SB. For all other models, no covariates were identified.

## Results

### Participant Characteristics

In total, 193 desk workers of companies from different industries (e.g. public service, automotive, education, social service, IT) were willing to participate starting in one of 15 sub-groups (4 sub-groups in intervention group 1, 2 sub-groups in intervention groups 2, 3 and 4, and 5 sub-groups in intervention group 5). Of the eligible participants, 43 declined before the kick-offs or did not create an account. Of the 150 participants with an account, 142 started with the baseline week (i.e. wore the VitaBit). During the intervention, 33 participants dropped out due to technical problems (*n* = 10), because they lost their device (*n* = 6), or due to other reasons, like time constraints (*n* = 17). Of the baseline participants, 109 participants (77%) stayed in the program until the end as indicated by still having data available and not having indicated to stop the intervention.

**Fig. 2:**
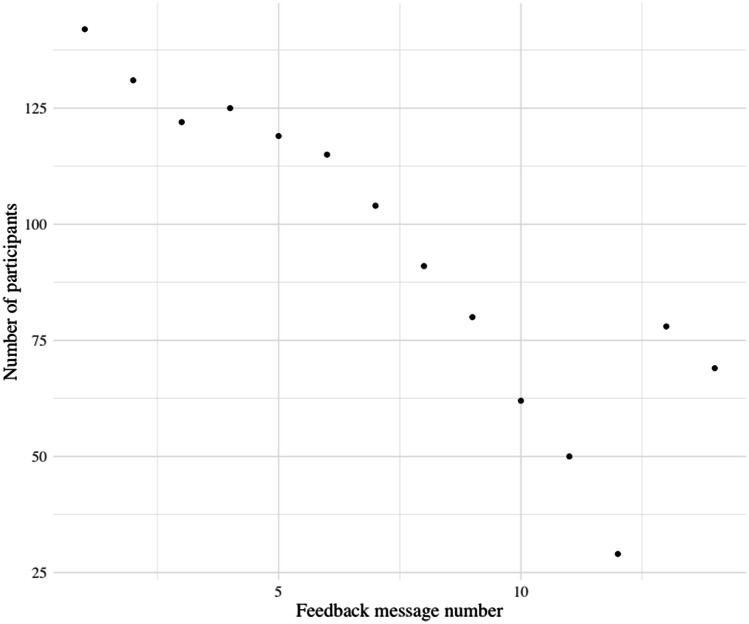
Number of participants having received specific feedback messages of UPcomplish

The survey was filled out by 129 (91%), 67 (47%) and 62 (44%) participants at T0, T1 and T2, respectively. The VitaBit was worn by 109 (75%) and 82 (56%) participants for at least 6 and 9 weeks, respectively. The number of people having received the n^th^ FBM decreased from FBM #1 (*n* = 141 [99% of the baseline participants]) to FBM #12 (*n* = 29, 20%). FBMs #13 (*n* = 78, 55%) and #14 (*n* = 69, 49%) were sent to all participants having data at the concerning points in time even if they had message delays. Figure 2 displays the number of participants having been sent FBMs to.

A total of 142 participants (96 females) wore the VitaBit device at baseline (Table 2). Participants had a median age of 42.0 (interquartile range [IQR] = 21.5) years and a mean BMI of 23.1 (standard deviation [SD] = 4.6) kg/m^2^. Of the participants who filled out the survey, the majority worked full-time and worked 5 days per week. At baseline, 63 (44%) participants met the program goal: maximally sitting for 8 h, minimally standing and walking for 4 h, and having a maximum of 18.8*10^3^ SSSB on at least 30% of the days (incl. weekend) [27].

**Table 2:** Descriptive characteristics of participants at baseline

	Female	Male	Total
	*n* = 96	*n* = 46	*n* = 142
Age (years), median (IQR)	41.0 (20.5)	44.0 (19.5)	42.0 (21.5)
Job-related variables			
Education level, *n* (%)^a^			
None	11 (11)	3 (7)	14 (10)
Secondary school	17 (18)	10 (22)	27 (19)
Professional	43 (45)	23 (50)	66 (46)
Work status, *n* (%)			
Full-time	65 (68)	39 (85)	104 (73)
Part-time	20 (21)	1 (2)	21 (15)
Workdays per week, *n* (%)			
4 workdays	7 (7)	2 (4)	9 (6)
5 workdays	76 (79)	37 (80)	113 (80)
6 workdays	0 (0)	3 (7)	3 (2)
Physical behaviour			
Wear time (min day^−1^), mean (SD)	835.7 (102.0)	797.8 (115.2)	823.4 (107.5)
Sedentary (min day^−1^), median (IQR)	504.4 (96.5)	522.3 (92.7)	510.2 (95.3)
Sedentary compositional geometric mean^c^, log-ratio variances standing, walking	62.3 (0.3, 0.2)	67.7 (0.2, 0.2)	64.3 (0.3, 0.2)
Standing (min day^−1^), median (IQR)	224.8 (129.7)	161.3 (73.2)	199.6 (102.8)
Standing compositional geometric mean^c^, log-ratio variances sitting, walking	27.2 (0.3, 0.2)	19.4 (0.2, 0.1)	24.5 (0.3, 0.3)
Activity (min day^−1^), median (IQR)	83.9 (45.6)	105.2 (37.8)	91.7 (45.7)
Activity compositional geometric mean^c^, log-ratio variances sitting, standing	10.5 (0.2, 0.2)	12.9 (0.2, 0.1)	11.3 (0.2, 0.3)
Program goal achieved (CER), *n* (%)	49 (51.0)	14 (30.4)	63 (44.4)
Anthropometrics^d^			
Height (cm)	168.6 (6.9)	180.5 (6.7)	172.4 (8.8)
Weight (kg)	65.0 (13.0)	80.0 (14.0)	69.0 (19.0)
BMI (kg/m^2^)	22.3 (5.1)	24.8 (3.5)	23.1 (4.6)
Underweight, *n* (%)	5 (5)	2 (4)	7 (5)
Normal weight, *n* (%)	46 (48)	14 (30)	60 (42)
Overweight, *n* (%)	16 (17)	14 (30)	30 (21)
Obese, *n* (%)	4 (4)	0 (0)	4 (3)

*SD* standard deviation, *IQR* interquartile range, min d^-1^ minutes per day, % d-1, proportion of the day, *CER* control event rate

^a^As indicated during the process of account creation

^b^Estimates of physical behaviours are estimated via VitaBit accelerometery. Control event rate: Maximum 8 hours sitting, minimum 4 hours standing and walking, 18.8*10³ SSSB on at least 30% of the days incl. weekend

^c^The percentage of the day is the estimated proportion of wearing-minutes spent in each activity level

^d^Underweight defines as BMI <18.5, Normal weight 18.5-25, overweight 25-30, obese > 30

### Effects of UPcomplish on SB

Between-subjects, UPcomplish did not result in a significant reduction of SB (Table 3). Within-subjects (Table 6, Appendix B), compared to baseline, participants were significantly more sedentary when they had received FBM #3 and #4.

**Table 3:** Multilevel linear models for the effects of different exposures to the UPcomplish intervention on SB

Intervention^a^	*n*^b^	SB CoDA		Summed squared sitting bouts	
		β (SE)	95% CI	β (SE)	95% CI
1	145 (116, 29)	− 0.01 (0.08)	− 0.16, 0.15	1.25 (6.68)	− 11.79, 14.29
Intercept		− 0.03 (0.07)	− 0.17, 0.12	− 5.50 (6.06)	− 17.33, 6.34
2	148 (107, 41)	− 0.01 (0.07)	− 0.14, 0.12	− 4.67 (5.15)	− 14.72, 5.38
Intercept		− 0.06 (0.06)	− 0.17, 0.06	− 8.35 (4.52)	− 17.18, 0.47
3	149 (90, 59)	0.01 (0.07)	− 0.12, 0.14	− 5.00 (5.32)	− 15.39, 5.38
Intercept		− 0.08 (0.06)	− 0.19, 0.02	− 8.44 (4.40)	− 17.03, 0.16
4	111 (74, 37)	− 0.02 (0.08)	− 0.17, 0.13	4.30 (6.87)	− 9.10, 17.70
Intercept		− 0.11 (0.06)	− 0.24, 0.02	− 11.67 (5.82)	− 23.02, − 0.32
5	114 (20, 94)	0.03 (0.07)	− 0.12, 0.18	3.98 (6.78)	− 9.77, 17.07
Intercept		− 0.05 (0.04)	− 0.12, 0.03	− 5.42 (3.73)	− 13.20, 1.72
6	126 (29, 97)	− 0.12 (0.07)	− 0.26, 0.03	− 9.26 (5.80)	− 21.07, 1.85
Intercept		− 0.03 (0.05)	− 0.12, 0.07	− 2.45 (3.78)	− 9.76, 5.19
7	141 (40, 101)	− 0.09 (0.06)	− 0.21, 0.03	− 0.34 (5.54)	− 11.05, 9.99
Intercept		− 0.03 (0.04)	− 0.10, 0.04	− 3.55 (3.56)	− 9.89, 3.23
8	134 (35, 99)	− 0.04 (0.06)	− 0.17, 0.07	− 3.57 (5.60)	− 14.90, 7.15
Intercept		− 0.01 (0.04)	− 0.08, 0.06	− 1.35 (3.87)	− 8.79, 6.56
9	119 (35, 84)	0.01 (0.07)	− 0.14, 0.14	− 3.05 (6.00)	− 15.25, 7.77
Intercept		− 0.02 (0.06)	− 0.10, 0.08	− 2.47 (4.65)	− 8.50, 6.20
10	96 (38, 58)	− 0.03 (0.08)	− 0.18, 0.11	− 0.28 (6.53)	− 13.23, 12.31
Intercept		− 0.04 (0.06)	− 0.16, 0.07	− 5.41 (5.47)	− 16.44, 5.13
11	45 (29, 16)	0.00 (0.12)	− 0.25, 0.24	5.82 (8.94)	− 13.16, 22.94
Intercept		− 0.12 (0.11)	− 0.32, 0.09	− 8.76 (12.50)	− 33.83, 17.22
12	18 (12, 6)	0.22 (0.26)	− 0.32, 0.66	17.24 (19.88)	− 23.27, 52.44
Intercept		− 0.05 (0.26)	− 0.55, 0.44	− 18.68 (19.55)	− 55.46, 18.19
13	8 (5, 3)	0.01 (0.16)	− 0.33, 0.31	− 13.49 (13.15)	− 38.78, 11.80
Intercept		0.05 (0.14)	− 0.22, 0.30	0.26 (10.40)	− 19.73, 20.25
14	80 (13, 67)	− 0.01 (0.09)	− 0.19, 0.16	− 1.22 (7.30)	− 15.42, 12.99
Intercept		− 0.03 (0.04)	− 0.11, 0.06	− 4.81 (3.57)	− 11.76, 2.14

For the multilevel linear models, the outcome variables were centred around the concerning baseline sub-group means. We adjusted for gender (locked in the model) and clustered by calendar week. Weeks where either no baseline or no respective feedback message was available, were excluded

*CI* confidence interval, *SE* standard error

^*^p < .05; **p < .01; ***p < .001 (after Benjamini–Hochberg correction)

^a^Feedback message is operationalized as having received this feedback message (and not more or less), which is compared to the baseline measurement of not having received any feedback

^b^Total number of observations (number of participants having received the concerning feedback message, number of participants at baseline being compared to)

### Effects of UPcomplish on QoL

Neither between-subjects (Table 4) nor within-subjects (Table 7, Appendix C) did the intervention reveal significant effects on QoL.

**Table 4:** Linear models for the effects of different exposures to the UPcomplish intervention on QoL

	Contextual performance			Task performance			Perceived stress		
Intervention^a^	*n*^b^	β (SE)	95% CI	*n*	β (SE)	95% CI	*n*	β (SE)	95% CI
1 to 5	49 (4, 45)	− 0.26 (0.30)	− 0.88, 0.35	48 (4, 44)	− 0.33 (0.27)	− 0.87, 0.22	48 (4, 44)	0.58 (2.89)	− 5.24, 6.40
Intercept		− 0.03 (0.09)	− 0.20, 0.15		− 0.02 (0.08)	− 0.18, 0.14		− 0.01 (0.83)	− 1.69, 1.67
6 to 8	101 (19, 82)	0.23 (0.14)	− 0.05, 0.52	100 (19, 81)	− 0.06 (0.15)	− 0.36, 0.24	100 (19, 81)	0.40 (1.56)	− 2.69, 3.50
Intercept		− 0.02 (0.06)	− 0.14, 0.10		− 0.02 (0.07)	− 0.15, 0.11		− 0.06 (0.68)	− 1.41, 1.29
9 to 11	100 (19, 81)	0.09 (0.14)	− 0.19, 0.36	99 (19, 80)	0.05 (0.14)	− 0.24, 0.33	99 (19, 80)	− 0.77 (1.54)	− 3.83, 2.28
Intercept		− 0.02 (0.06)	− 0.14, 0.10		− 0.02 (0.06)	− 0.15, 0.11		− 0.09 (0.67)	− 1.43, 1.25
12 to 14	79 (15, 64)	0.05 (0.16)	− 0.28, 0.37	78 (15, 63)	0.08 (0.16)	− 0.23, 0.40	78 (15, 63)	− 1.49 (1.79)	− 5.05, 2.07
Intercept		− 0.02 (0.07)	− 0.16, 0.12		− 0.02 (0.07)	− 0.16, 0.12		0.04 (0.78)	− 1.52, 1.60
	Perceived pain (inverse)^c^			Vitality			Emotional well-being		
1 to 5	47 (4, 43)	4.26 (12.09)	− 20.09, 28.61	47 (4, 43)	− 2.04 (9.40)	− 20.98, 16.90	47 (4, 43)	− 2.53 (6.47)	− 15.57, 10.51
Intercept		− 1.19 (3.53)	− 8.29, 5.91		− 0.90 (2.74)	− 6.43, 4.62		− 0.37 (1.89)	− 4.17, 3.44
6 to 8	99 (19, 80)	1.57 (5.51)	− 9.36, 12.51	99 (19, 80)	2.88 (4.92)	− 6.88, 12.64	99 (19, 80)	0.07 (3.53)	− 6.92, 7.07
Intercept		− 0.35 (2.41)	− 5.14, 4.44		− 0.15 (2.15)	− 4.42, 4.13		− 0.07 (1.54)	− 3.14, 2.99
9 to 11	97 (18, 79)	− 0.04 (6.17)	− 12.30, 12.21	97 (18, 79)	6.28 (5.16)	− 3.96, 16.51	97 (18, 79)	3.97 (3.59)	− 3.16, 11.10
Intercept		− 0.57 (2.66)	− 5.85, 4.71		− 0.48 (2.22)	− 4.89, 3.93		− 0.04 (1.55)	− 3.11, 3.03
12 to 14	77 (15, 62)	11.90 (6.45)	− 0.95, 24.76	77 (15, 62)	9.79 (5.68)	− 1.53, 21.11	77 (15, 62)	4.26 (4.33)	− 4.37, 12.90
Intercept		− 0.92 (2.85)	− 6.59, 4.76		− 0.60 (2.51)	− 5.60, 4.39		− 0.19 (1.91)	− 4.00, 3.62

For the linear models, the outcome variables were centred around the baseline sub-group means. Due to singularity, the models were not clustered by calendar weeks. After backwards elimination, no covariates were included. Weeks where either no baseline or no respective feedback message was available, were excluded

*CI* confidence interval, *SE* standard error

^*^p < .05; **p < .01; ***p < .001 (after Benjamini–Hochberg correction)

^a^Feedback message is operationalized as having received this feedback message (and not more or less), which is compared to the baseline measurement of not having received any feedback

^b^Total number of observations (number of participants having received the concerning feedback message, number of participants at baseline being compared to)

^c^Perceived pain is inverted, i.e. higher numbers refers to not having any physical complaints

### Effects of UPcomplish on Psychosocial Determinants

Participants having received FBMs number #6, #7, or #8 reported significantly lower PS compared to baseline (Table 5). Within-subjects (Appendix D, Table 8) compared to baseline, after having received FBMs number #6, #7, or #8, they reported significantly lower PS, and after having received FBMs number #1 to #5, significantly higher intentions to reduce their SB.

**Table 5:** Linear models for the effects of different exposures to the UPcomplish intervention on psychosocial determinants

		Attitude		Perceived social support		Perceived behavioural control		Perceived Susceptibility		Intention	
Intervention^a^	*n*^b^	β (SE)	95% CI	β (SE)	95% CI	β (SE)	95% CI	β (SE)	95% CI	β (SE)	95% CI
1 to 5	49 (4, 45)	0.11 (0.24)	− 0.37, 0.58	0.29 (0.42)	− 0.55, 1.12	− 0.14 (0.33)	− 0.81, 0.52	0.01 (0.37)	− 0.73, 0.76	− 0.18 (0.45)	− 1.08, 0.72
Intercept		− 0.02 (0.07)	− 0.16, 0.11	0.04 (0.12)	− 0.20, 0.27	− 0.03 (0.09)	− 0.21, 0.16	− 0.03 (0.11)	− 0.24, 0.18	0.01 (0.13)	− 0.24, 0.27
6 to 8	101 (19, 82)	0.00 (0.12)	− 0.24, 0.24	− 0.03 (0.22)	− 0.47, 0.41	0.16 (0.16)	− 0.16, 0.47	− 0.66 (0.18)*	− 1.03, − 0.30	0.20 (0.23)	− 0.26, 0.65
Intercept		0.01 (0.05)	− 0.09, 0.12	0.00 (0.1)	− 0.19, 0.19	0.00 (0.07)	− 0.14, 0.14	− 0.03 (0.08)	− 0.18, 0.13	0.04 (0.1)	− 0.16, 0.23
9 to 11	100 (19, 81)	− 0.04 (0.11)	− 0.27, 0.19	0.05 (0.2)	− 0.35, 0.45	− 0.08 (0.16)	− 0.39, 0.24	− 0.30 (0.18)	− 0.66, 0.07	− 0.06 (0.21)	− 0.47, 0.36
Intercept		0.01 (0.05)	− 0.09, 0.10	0.00 (0.09)	− 0.17, 0.18	− 0.01 (0.07)	− 0.15, 0.13	− 0.03 (0.08)	− 0.19, 0.13	0.03 (0.09)	− 0.15, 0.21
12 to 14	79 (15, 64)	0.09 (0.15)	− 0.21, 0.39	0.13 (0.22)	− 0.31, 0.58	0.08 (0.2)	− 0.31, 0.47	− 0.62 (0.26)	− 1.14, − 0.10	0.04 (0.26)	− 0.48, 0.56
Intercept		− 0.01 (0.07)	− 0.14, 0.12	0.05 (0.1)	− 0.15, 0.24	− 0.01 (0.09)	− 0.18, 0.16	− 0.03 (0.11)	− 0.25, 0.20	0.03 (0.11)	− 0.20, 0.25

For the linear models, the outcome variables were centred around the baseline sub-group means. Due to singularity, the models were not clustered by calendar weeks. After backwards elimination, no covariates were included. Weeks where either no baseline or no respective feedback message was available, were excluded

*CI* confidence interval, *SE* standard error

^*^p < .05; **p < .01; ***p < .001 (after Benjamini–Hochberg correction)

^a^Feedback message is operationalized as having received this feedback message (and not more or less), which is compared to the baseline measurement of not having received any feedback

^b^Total number of observations (number of participants having received the concerning feedback message, number of participants at baseline being compared to)

### Post Hoc Analyses

**Fig. 3:**
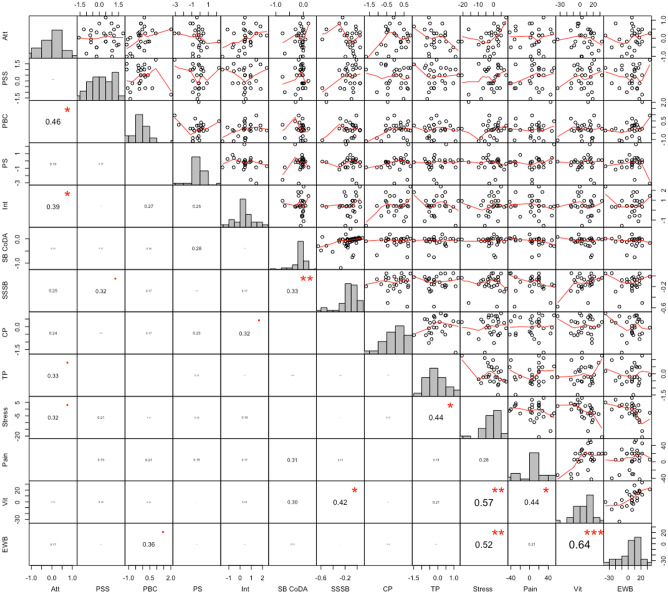
Pearson correlations and plots illustrating the linear and smoothed associations, respectively, between improvements in determinants (T2–T0), SB (week to week) and QoL (T2–T0). Abbreviations: Att, attitude; PSS, perceived social support; PBC, perceived behavioural control; PS, perceived susceptibility; Int, intention; SB CoDA, SB proportion; SSSB, summed squared sitting bouts; CP, contextual performance; TP, task performance; Stress, perceived stress; Pain, not having any pain; Vit, perceived vitality; EWB, emotional well-being

Figure 3 shows the correlations as well as univariate distributions of the variables. Improvement refers to a beneficial development from worse values in the beginning (e.g. more perceived stress, more sitting, less performance) to better values in the end. Firstly, we analysed the correlations of the variables within the clusters (i.e., psychosocial determinants, SB variables, and QoL variables). After Benjamini–Hochberg correction, the improvement of vitality was positively associated with improvement of stress (*r* = 0.57; 95% CI = 0.25, 0.78; *p* < 0.01; *p*_corrected_ = 0.02) and EWB (*r* = 0.64; 95% CI = 0.35, 0.82; *p* = *p*_corrected_ < 0.001). Secondly, we studied correlations of improvements in the psychosocial determinants with improvements in SB, which were not found. Thirdly, we examined correlations of improvements in SB with improvements in QoL, which were not found.

## Discussion

This study investigated whether receiving UPcomplish had an effect on SB, QoL and psychosocial determinants as compared to VitaBit only phases. The results suggest that neither on SB nor on QoL does the 12-week intervention have beneficial effects when compared to VitaBit only phases. When having received FBMs #6, #7 or #8, the participants felt less susceptible compared to both their own baseline and others at their baseline, i.e. they agreed less that they should reduce their SB. When having received 5 or fewer FBMs, they indicated higher intentions to reduce and regularly interrupt SB compared to their baseline. None of the improvements in psychosocial determinants was associated with improvements in SB, and improvements in SB were not associated with improvements in QoL.

These results are in line with the evaluations of persuasion only interventions that did not reveal SB reductions [34, 58, 59], notwithstanding any publication bias [60]. Although the relative distribution of the time spent sitting at baseline (64.3%) was similar to the distributions that were previously found among office workers [61, 62], already 44% of the study population met the program goals that had been formulated for the intervention [27]. First, wearing a monitoring device and having received health information during the kick-offs might already have had positive effects [36, 63]. Second, the voluntary participation might have resulted in a selection bias that only participants being interested in a healthy lifestyle participated [64]. This was reflected by positive baseline attitudes towards reducing SB, high baseline QoL, and by the low response rate. Indeed, there seems to be a tendency that interventions with target groups showing more SB at baseline have greater effects on SB compared to target groups with fewer SB [34]. Post hoc analyses within the scope of moderation analyses (in preparation) investigating differences between the participants with more and the ones with less baseline SB are therefore warranted [27, 29].

Several aspects might have impeded the effectiveness of the intervention. Firstly, while environmental changes such as standing desks have been found to be helpful [18, 34, 36], the individual employee possesses limited possibilities to reduce sitting at work due to, for example, time constraints and the ways that work is structured (e.g. lengthy meetings at round-tables). Additionally, SB has become a habitual process because it is linked to diverse contexts and activities, such as work, and standing at work is perceived as uncomfortable [65–67]. Unsurprisingly, therefore, that others only found significant SB reductions on weekends [20, 26]. SB might be less of a reasoned behaviour and more determined by environmental, societal, or habitual factors. This is also reflected in the fact that none of the improvements in psychosocial determinants was associated with improvements in SB. Secondly, due to the high baseline QoL values and the selectivity of the sample [64], short-term effects of this intervention such as reductions of back-pain or an increase of vitality might not have been as dominant in order to serve as additional motivators [68], which was also reflected by the poor correlations between improvements in SB and QoL. Thirdly, the FBMs of this intervention merely focused on workplace SB. Since this constitutes the majority of the daily life, we had expected an overall reduction of SB. Nevertheless, while it might have had a reducing effect only on workplace sitting, leisure time habits (after working hours and weekends) might have mitigated the effects, which was already found in other SB interventions focusing on workplace sitting [24, 25]. Lastly, there seems to be a tendency that the intervention reduced SB after 6 to 8 FBMs, but the perceived need to sit less (i.e. PS) also drops around this moment. This might have been a reason why higher intentions to reduce SB after the first few FBMs were not stable. To keep participants motivated, they were provided with a lot of positive feedback on their progress. Additional to positive feedback, it might, hence, be important to still remind participants of their initial goals and general SB recommendations. Blom et al. evaluated two personal counselling interventions and only found an increase in controlled and autonomous motivation after the physical activity intervention and not after the SB intervention [67]. It might be helpful to have another personal meeting in the middle of the intervention, to keep sending two FBMs per week, or to adapt the FBMs themselves, also with a greater focus on healthy alternative behaviours and reminding of SB recommendations.

### Strengths and Limitations

The current study examined the effects that a workplace SB intervention has on overall daily sitting. While many interventions only analyse the effects on workplace sitting, this study focuses on the target group and their entire daily life providing better external validity and a valid predictor for participants’ general health. Furthermore, the analyses respected the compositional and inter-dependent nature of physical behaviours, and included a novel, yet intuitive, operationalization of prolonged sitting. Additionally, the drop-out rates were smaller compared to other workplace physical activity and SB interventions, and they were mostly due to technical problems rather than a loss of motivation [69]. This is an indicator of the acceptability and, thereby, the potential of the UPcomplish intervention. Lastly, the stepped-wedge design revealed more data points and reliability per participant, and allowed for high external validity since data were collected throughout 75% of the year.

The study also has some limitations. Firstly, we assume a recruitment bias among participants which might have resulted in a group of participants being dominated by females, being healthier and more motivated than the average office employee. Nonetheless, baseline SB proportions of participants are comparable to what was found in previous studies. Secondly, we included participants of diverse workplace sites and with potential underlying health problems (e.g. cardiac problems), which might not be comparable in terms of SB and the potential to reduce it. Nonetheless to increase internal and external validity, we centred all outcome variables around baseline company means, and included multiple company industries, education levels, team and company sizes. Additionally, we not only conducted between-subjects but also within-subjects statistical analyses which increased statistical power and generalizability of the results. Similarly, since all FBMs of the intervention were tailored, the effects might not be fully comparable across subjects. However, all FBMs were drafted based on the change objectives and the parameters of effectiveness of the UPcomplish intervention. This ensured that each participant received FBMs relevant to them, which is one of the requirements for the methods to be effective in behaviour change. Lastly, the kick-off session prior to the UPcomplish intervention could be considered as an additional intervention. Yet, previous research did not find effects of providing generic information on SB and its consequences and this study investigated the effects of providing tailored feedback.

This study provides an essential addition to the literature on SB. Although UPcomplish was structurally developed using evidence from the literature and from theory, it was neither effective in improving SB nor QoL. In the middle of the intervention, participants perceived to be less susceptible to being sedentary. Firstly, we conclude that a workplace SB intervention might need to focus more on structural changes of the workplace environment. Secondly, workplace sitting might not only be influenced by the psychosocial determinants that were chosen for this intervention, but also by other psychosocial determinants or by different underlying beliefs. Lastly, the sample seemed to be selective in such a way that the participants were likely more motivated, less sedentary and had higher QoL compared to the average office worker. In an additional study, potential moderators of the effectiveness of UPcomplish will be investigated to explore whether UPcomplish was effective for certain subgroups, such as people being more sedentary.

## Supplementary Information

Below is the link to the electronic supplementary material.

Supplementary file1 (DOCX 43 KB)

Supplementary file2 (DOCX 24 KB)

Supplementary file3 (DOCX 19 KB)

Supplementary file4 (DOCX 17 KB)

## Data Availability

The cleaned raw data and additional material is fully disclosed in the supplementary materials (https://osf.io/qzp9m/?view_only=30ada8d6fc0e4ac19a1610b8901f9f96).

## Abbreviations

SB: Sedentary behaviours
QoL: Quality of life
FBM: Feedback message
MVPA: Moderate-to-vigorous physical activity
IM: Intervention mapping

## Footnotes

1 Although the intervention and the advice focused on the reduction of SB (e.g. drink more to sit less), participants could also set a physical activity goal (e.g. being active for at least 1 h per day), which enabled a provision of choice. Participants with physical activity goals received similar feedback on SB patterns (e.g. your sitting bouts are the longest on Tuesday mornings) but different feedback on the achievement of goals (e.g. you reached your goal of moving at least 1 h on Wednesday).
