# Supplementary figures and images for: The Effects of UPcomplish on Office Workers’ Sedentary Behaviour, Quality of Life and Psychosocial Determinants: A Stepped-Wedge Design
Source: Int J Behav Med. 2022 Jan 31;29(6):728–42. doi: 10.1007/s12529-022-10054-0 (PMC9684295; doi:10.1007/s12529-022-10054-0)

Appendix *A*


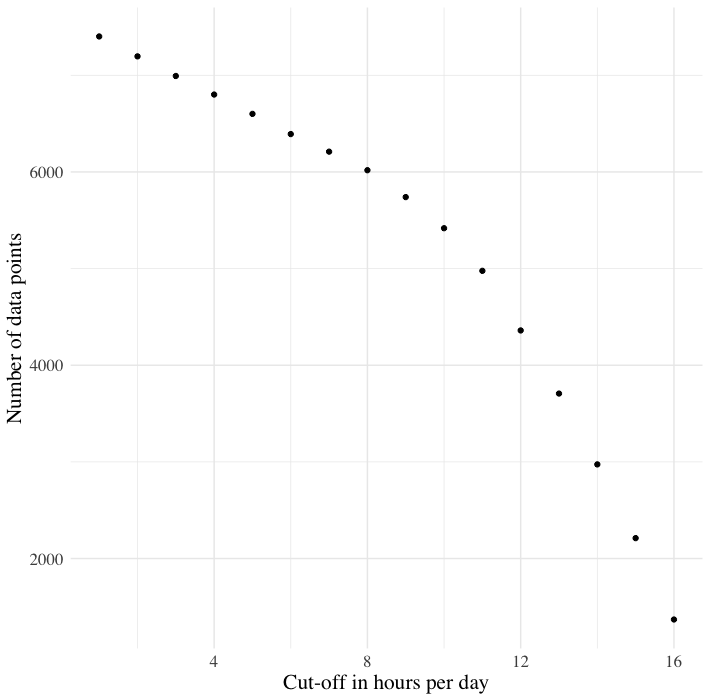


Figure 2.

Supplement: Supplementary file 1 — Supplementary file1 (DOCX 43 KB) [file 12529_2022_10054_MOESM1_ESM.docx]
