# Supplementary material for: The Effects of UPcomplish on Office Workers’ Sedentary Behaviour, Quality of Life and Psychosocial Determinants: A Stepped-Wedge Design
Source: Int J Behav Med. 2022 Jan 31;29(6):728–42. doi: 10.1007/s12529-022-10054-0 (PMC9684295; doi:10.1007/s12529-022-10054-0)
Supplement: Supplementary file 2 — Supplementary file2 (DOCX 24 KB) [file 12529_2022_10054_MOESM2_ESM.docx]

### Appendix B

| **Table 6.** Multilevel linear models for the effects of different exposures to the UPcomplish intervention on SB parameters ^a^ (random intercept on the individual level) | | | | | |
| --- | --- | --- | --- | --- | --- |
|  |  | *SB CoDA* | | *Summed Squared Sitting Bouts* | |
| Intervention *^b^* | n | β *(SE)* | 95% CI | β *(SE)* | 95% CI |
| 1 | 236 | -0.03 (0.03) | -0.10, 0.03 | -0.06 (3.61) | -7.15, 7.06 |
| Intercept |  | -0.01 (0.03) | -0.08, 0.06 | 0.52 (3.13) | -5.62, 6.65 |
| 2 | 224 | 0.01 (0.04) | -0.07, 0.08 | -0.50 (3.68) | -7.74, 6.75 |
| Intercept |  | -0.02 (0.04) | -0.09, 0.05 | -0.87 (2.96) | -6.68, 4.94 |
| 3 | 197 | 0.24 (0.05)*** | 0.15, 0.33 | 20.83 (3.53)*** | 13.90, 27.78 |
| Intercept |  | -0.01 (0.04) | -0.08, 0.07 | -1.09 (2.95) | -6.87, 4.69 |
| 4 | 186 | 0.20 (0.05)*** | 0.11, 0.29 | 24.80 (4.56)*** | 15.84, 33.76 |
| Intercept |  | -0.01 (0.04) | -0.08, 0.06 | -0.49 (3.14) | -6.66, 5.67 |
| 5 | 129 | 0.05 (0.07) | -0.09, 0.18 | 2.37 (6.92) | -11.40, 15.94 |
| Intercept |  | -0.01 (0.03) | -0.08, 0.05 | -0.05 (2.75) | -5.44, 5.34 |
| 6 | 129 | -0.08 (0.07) | -0.21, 0.06 | -11.30 (6.38) | -23.79, 1.19 |
| Intercept |  | -0.03 (0.04) | -0.10, 0.04 | 0.17 (3.02) | -5.76, 6.09 |
| 7 | 135 | -0.08 (0.07) | -0.21, 0.05 | 0.03 (5.89) | -11.52, 11.58 |
| Intercept |  | -0.01 (0.04) | -0.08, 0.07 | 1.55 (3.21) | -4.73, 7.84 |
| 8 | 113 | 0.04 (0.07) | -0.09, 0.17 | 4.82 (5.62) | -6.20, 16.06 |
| Intercept |  | -0.02 (0.04) | -0.10, 0.06 | 0.69 (3.37) | -5.91, 7.30 |
| 9 | 105 | 0.05 (0.07) | -0.09, 0.19 | -2.04 (6.04) | -13.94, 9.89 |
| Intercept |  | -0.02 (0.05) | -0.12, 0.07 | 0.70 (3.89) | -6.93, 8.33 |
| 10 | 95 | 0.10 (0.07) | -0.05, 0.24 | 6.48 (6.26) | -5.80, 18.98 |
| Intercept |  | -0.04 (0.06) | -0.15, 0.07 | 0.46 (4.49) | -8.34, 9.27 |
| 11 | 76 | 0.17 (0.08) | 0.02, 0.33 | 9.62 (6.81) | -3.85, 23.13 |
| Intercept |  | -0.05 (0.06) | -0.17, 0.06 | -1.07 (4.81) | -10.51, 8.37 |
| 12 | 38 | 0.31 (0.13) | 0.06, 0.57 | 19.97 (10.23) | -0.08, 40.19 |
| Intercept |  | -0.03 (0.08) | -0.18, 0.12 | -0.86 (5.79) | -12.19, 10.46 |
| 13 | 58 | 0.00 (0.1) | -0.21, 0.20 | -29.37 (9.4) | -48.28, -10.02 |
| Intercept |  | -0.03 (0.04) | -0.11, 0.06 | 1.08 (3.62) | -6.04, 8.21 |
| 14 | 53 | -0.04 (0.1) | -0.24, 0.17 | -12.33 (8.91) | -29.82, 5.45 |
| Intercept |  | -0.07 (0.06) | -0.18, 0.04 | 0.13 (4.69) | -9.05, 9.32 |
| Abbreviations: CI, confidence interval; SE, standard error.  ^a^ For the multilevel linear models, the outcome variables were centred around the baseline calendar week means. The models were clustered by individuals. After backwards elimination, no covariates were included.  ^b^ Feedback message is operationalized as having received this feedback message (and not more or less), which is compared to the baseline measurement of not having received any feedback.  *** *p* < .001; ** *p* < .01; * *p* < .05 (after Benjamini-Hochberg correction) | | | | | |

### Appendix B

| **Table 6.** Multilevel linear models for the effects of different exposures to the UPcomplish intervention on SB parameters ^a^ (random intercept on the individual level) | | | | | |
| --- | --- | --- | --- | --- | --- |
|  |  | *SB CoDA* | | *Summed Squared Sitting Bouts* | |
| Intervention *^b^* | n | β *(SE)* | 95% CI | β *(SE)* | 95% CI |
| 1 | 236 | -0.03 (0.03) | -0.10, 0.03 | -0.06 (3.61) | -7.15, 7.06 |
| Intercept |  | -0.01 (0.03) | -0.08, 0.06 | 0.52 (3.13) | -5.62, 6.65 |
| 2 | 224 | 0.01 (0.04) | -0.07, 0.08 | -0.50 (3.68) | -7.74, 6.75 |
| Intercept |  | -0.02 (0.04) | -0.09, 0.05 | -0.87 (2.96) | -6.68, 4.94 |
| 3 | 197 | 0.24 (0.05)*** | 0.15, 0.33 | 20.83 (3.53)*** | 13.90, 27.78 |
| Intercept |  | -0.01 (0.04) | -0.08, 0.07 | -1.09 (2.95) | -6.87, 4.69 |
| 4 | 186 | 0.20 (0.05)*** | 0.11, 0.29 | 24.80 (4.56)*** | 15.84, 33.76 |
| Intercept |  | -0.01 (0.04) | -0.08, 0.06 | -0.49 (3.14) | -6.66, 5.67 |
| 5 | 129 | 0.05 (0.07) | -0.09, 0.18 | 2.37 (6.92) | -11.40, 15.94 |
| Intercept |  | -0.01 (0.03) | -0.08, 0.05 | -0.05 (2.75) | -5.44, 5.34 |
| 6 | 129 | -0.08 (0.07) | -0.21, 0.06 | -11.30 (6.38) | -23.79, 1.19 |
| Intercept |  | -0.03 (0.04) | -0.10, 0.04 | 0.17 (3.02) | -5.76, 6.09 |
| 7 | 135 | -0.08 (0.07) | -0.21, 0.05 | 0.03 (5.89) | -11.52, 11.58 |
| Intercept |  | -0.01 (0.04) | -0.08, 0.07 | 1.55 (3.21) | -4.73, 7.84 |
| 8 | 113 | 0.04 (0.07) | -0.09, 0.17 | 4.82 (5.62) | -6.20, 16.06 |
| Intercept |  | -0.02 (0.04) | -0.10, 0.06 | 0.69 (3.37) | -5.91, 7.30 |
| 9 | 105 | 0.05 (0.07) | -0.09, 0.19 | -2.04 (6.04) | -13.94, 9.89 |
| Intercept |  | -0.02 (0.05) | -0.12, 0.07 | 0.70 (3.89) | -6.93, 8.33 |
| 10 | 95 | 0.10 (0.07) | -0.05, 0.24 | 6.48 (6.26) | -5.80, 18.98 |
| Intercept |  | -0.04 (0.06) | -0.15, 0.07 | 0.46 (4.49) | -8.34, 9.27 |
| 11 | 76 | 0.17 (0.08) | 0.02, 0.33 | 9.62 (6.81) | -3.85, 23.13 |
| Intercept |  | -0.05 (0.06) | -0.17, 0.06 | -1.07 (4.81) | -10.51, 8.37 |
| 12 | 38 | 0.31 (0.13) | 0.06, 0.57 | 19.97 (10.23) | -0.08, 40.19 |
| Intercept |  | -0.03 (0.08) | -0.18, 0.12 | -0.86 (5.79) | -12.19, 10.46 |
| 13 | 58 | 0.00 (0.1) | -0.21, 0.20 | -29.37 (9.4) | -48.28, -10.02 |
| Intercept |  | -0.03 (0.04) | -0.11, 0.06 | 1.08 (3.62) | -6.04, 8.21 |
| 14 | 53 | -0.04 (0.1) | -0.24, 0.17 | -12.33 (8.91) | -29.82, 5.45 |
| Intercept |  | -0.07 (0.06) | -0.18, 0.04 | 0.13 (4.69) | -9.05, 9.32 |
| Abbreviations: CI, confidence interval; SE, standard error.  ^a^ For the multilevel linear models, the outcome variables were centred around the baseline calendar week means. The models were clustered by individuals. After backwards elimination, no covariates were included.  ^b^ Feedback message is operationalized as having received this feedback message (and not more or less), which is compared to the baseline measurement of not having received any feedback.  *** *p* < .001; ** *p* < .01; * *p* < .05 (after Benjamini-Hochberg correction) | | | | | |
