# Supplementary material for: The Effects of UPcomplish on Office Workers’ Sedentary Behaviour, Quality of Life and Psychosocial Determinants: A Stepped-Wedge Design
Source: Int J Behav Med. 2022 Jan 31;29(6):728–42. doi: 10.1007/s12529-022-10054-0 (PMC9684295; doi:10.1007/s12529-022-10054-0)
Supplement: Supplementary file 3 — Supplementary file3 (DOCX 19 KB) [file 12529_2022_10054_MOESM3_ESM.docx]

| Appendix C **Table 7.** Multilevel linear models for the effects of different exposures to the UPcomplish intervention on QoL (random intercept on the individual level) ^a^ | | | | | | | | | |
| --- | --- | --- | --- | --- | --- | --- | --- | --- | --- |
|  | *Contextual performance* | | | *Task performance* | | | *Perceived stress* | | |
| Intervention ^b^ | *n* | β *(SE)* | 95% CI | *n* | β *(SE)* | 95% CI | *n* | β *(SE)* | 95% CI |
| 1 to 5 | *16* | -0.34 (0.31) | -0.96, 0.27 | *16* | -0.63 (0.42) | -1.45, 0.19 | *16* | 1.70 (2.98) | -4.13, 8.34 |
| Intercept |  | 0.04 (0.18) | -0.31, 0.38 |  | -0.01 (0.21) | -0.42, 0.40 |  | 1.89 (1.84) | -1.75, 5.53 |
| 6 to 8 | *57* | -0.02 (0.14) | -0.30, 0.26 | *56* | -0.20 (0.15) | -0.51, 0.11 | *56* | 2.60 (1.56) | -0.61, 5.65 |
| Intercept |  | -0.06 (0.1) | -0.26, 0.15 |  | -0.07 (0.12) | -0.31, 0.16 |  | -0.60 (1.09) | -2.74, 1.52 |
| 9 to 11 | *58* | 0.07 (0.12) | -0.15, 0.31 | *58* | -0.07 (0.13) | -0.32, 0.19 | *58* | -0.86 (1.02) | -2.88, 1.22 |
| Intercept |  | -0.04 (0.08) | -0.20, 0.13 |  | 0.02 (0.09) | -0.15, 0.20 |  | 0.28 (1.07) | -1.85, 2.40 |
| 12 to 14 | *45* | 0.03 (0.15) | -0.28, 0.33 | *45* | 0.06 (0.16) | -0.28, 0.38 | *45* | -2.31 (1.61) | -5.50, 1.00 |
| Intercept |  | -0.11 (0.12) | -0.35, 0.13 |  | -0.10 (0.12) | -0.34, 0.14 |  | 0.35 (1.2) | -2.02, 2.72 |
|  | *Perceived pain (inverse)*^c^ | | | *Vitality* | | | *Emotional well-being* | | |
| 1 to 5 | *16* | 5.77 (12.05) | -17.80, 34.60 | *16* | 0.51 (6.66) | -13.31, 14.31 | *16* | 1.76 (6.27) | -14.29, 14.23 |
| Intercept |  | -13.56 (7.02) | -27.38, 0.26 |  | -6.91 (4.87) | -16.61, 2.79 |  | -6.86 (3.65) | -14.06, 0.33 |
| 6 to 8 | *56* | 2.65 (5.31) | -7.78, 13.49 | *56* | -5.51 (3.8) | -12.96, 2.38 | *56* | -4.06 (3.65) | -11.21, 3.52 |
| Intercept |  | 4.38 (3.51) | -2.50, 11.27 |  | 2.43 (3.03) | -3.53, 8.42 |  | 0.99 (2.49) | -3.90, 5.89 |
| 9 to 11 | *57* | -0.90 (5.85) | -12.61, 10.64 | *57* | 6.53 (2.79) | 0.92, 12.08 | *57* | 2.53 (2.56) | -2.58, 7.64 |
| Intercept |  | -0.16 (4.21) | -8.44, 8.12 |  | -0.93 (3.23) | -7.31, 5.45 |  | 0.33 (2.25) | -4.11, 4.77 |
| 12 to 14 | *45* | 10.68 (5.47) | -0.05, 21.97 | *45* | 7.82 (4.62) | -1.43, 17.21 | *45* | 5.70 (3.85) | -2.09, 13.37 |
| Intercept |  | 6.40 (3.83) | -1.12, 13.92 |  | -0.01 (3.61) | -7.14, 7.12 |  | -0.26 (2.95) | -6.07, 5.56 |
| Abbreviations: CI, confidence interval; SE, standard error.  ^a^ For the multilevel linear models, the outcome variables were centred around the baseline calendar week means. The models were clustered by individuals. After backwards elimination, no covariates were included.  ^b^ Feedback message is operationalized as having received this feedback message (and not more or less), which is compared to the baseline measurement of not having received any feedback.  ^c^ Perceived pain is inverted, i.e. higher numbers refers to not having any physical complaints.  *** *p* < .001; ** *p* < .01; * *p* < .05 (after Benjamini-Hochberg correction) | | | | | | | | | |
