# Supplementary material for: The Effects of UPcomplish on Office Workers’ Sedentary Behaviour, Quality of Life and Psychosocial Determinants: A Stepped-Wedge Design
Source: Int J Behav Med. 2022 Jan 31;29(6):728–42. doi: 10.1007/s12529-022-10054-0 (PMC9684295; doi:10.1007/s12529-022-10054-0)
Supplement: Supplementary file 4 — Supplementary file4 (DOCX 17 KB) [file 12529_2022_10054_MOESM4_ESM.docx]

### Appendix D

| **Table 8.** Multilevel linear models for the effects of different exposures to the UPcomplish intervention on psychosocial determinants (random intercept on the individual level) ^a^ | | | | | | | | | | | |
| --- | --- | --- | --- | --- | --- | --- | --- | --- | --- | --- | --- |
|  | | *Attitude* | | *Perceived social support* | | *Perceived behavioural control* | | *Perceived Susceptibility* | | *Intention* | |
| Intervention ^b^ | *n* | β *(SE)* | 95% CI | β *(SE)* | 95% CI | β *(SE)* | 95% CI | β *(SE)* | 95% CI | β *(SE)* | 95% CI |
| 1 to 5 | *16* | 0.81 (0.24) | 0.32, 1.33 | 1.30 (0.43) | 0.45, 2.20 | 0.49 (0.19) | 0.10, 0.89 | -0.16 (0.28) | -0.74, 0.47 | 1.14 (0.27)* | 0.61, 1.66 |
| Intercept |  | -0.13 (0.17) | -0.46, 0.20 | 0.32 (0.3) | -0.28, 0.91 | -0.27 (0.18) | -0.64, 0.11 | -0.30 (0.24) | -0.79, 0.19 | -0.09 (0.13) | -0.35, 0.17 |
| 6 to 8 | *57* | 0.17 (0.13) | -0.08, 0.43 | 0.25 (0.22) | -0.18, 0.68 | 0.17 (0.16) | -0.16, 0.49 | -0.75 (0.22)* | -1.18, -0.32 | 0.42 (0.24) | -0.06, 0.90 |
| Intercept |  | -0.07 (0.1) | -0.25, 0.12 | 0.02 (0.14) | -0.25, 0.30 | -0.02 (0.12) | -0.26, 0.21 | -0.03 (0.13) | -0.28, 0.22 | 0.02 (0.17) | -0.32, 0.36 |
| 9 to 11 | *58* | 0.08 (0.13) | -0.17, 0.36 | 0.59 (0.2) | 0.20, 0.99 | -0.05 (0.12) | -0.28, 0.18 | -0.44 (0.18) | -0.80, -0.09 | 0.29 (0.22) | -0.17, 0.73 |
| Intercept |  | -0.01 (0.08) | -0.18, 0.15 | -0.04 (0.11) | -0.27, 0.18 | 0.01 (0.1) | -0.18, 0.20 | 0.05 (0.13) | -0.20, 0.30 | 0.12 (0.14) | -0.15, 0.38 |
| 12 to 14 | *45* | -0.11 (0.14) | -0.38, 0.16 | 0.34 (0.2) | -0.07, 0.74 | -0.23 (0.14) | -0.52, 0.05 | -0.44 (0.24) | -0.91, 0.04 | 0.24 (0.23) | -0.22, 0.71 |
| Intercept |  | 0.06 (0.1) | -0.14, 0.26 | -0.14 (0.16) | -0.46, 0.17 | 0.04 (0.12) | -0.21, 0.28 | 0.15 (0.15) | -0.14, 0.44 | 0.09 (0.17) | -0.25, 0.42 |
| Abbreviations: CI, confidence interval; SE, standard error.  ^a^ For the multilevel linear models, the outcome variables were centred around the baseline calendar week means. The models were clustered by individuals. After backwards elimination, no covariates were included.  ^b^ Feedback message is operationalized as having received this feedback message (and not more or less), which is compared to the baseline measurement of not having received any feedback.  *** *p* < .001; ** *p* < .01; * *p* < .05 (after Benjamini-Hochberg correction) | | | | | | | | | | | |
